# The clinical value of inflammatory factors in evaluating the prognosis of patients with acute myeloid leukemia

**DOI:** 10.5937/jomb0-57965

**Published:** 2025-11-05

**Authors:** Jianghuizi Li, Feng Liu, Wen Wu

**Affiliations:** 1 Xian Central Hospital/Xian Institute of Hematology, Department of Hematology, Xian, Shaanxi, China

**Keywords:** acute myeloid leukemia, prognosis, TNF-a, LIF, IFNg, IL-2, IL-4, IL-17A, akutna mijeloidna leukemija, prognoza, TNF-a, LIF, IFNg, IL-2, IL-4, IL-17A

## Abstract

**Background:**

To assess the importance of inflammatory factors in predicting outcomes in individuals diagnosed with acute myeloid leukemia.

**Methods:**

Between July 2017 and December 2019, a total of 100 patients with a recent diagnosis of acute myeloid leukemia (AML) were recruited from the Hematology Department of our hospital's Cancer Center and assigned to the AML group. Additionally, 60 individuals with no underlying health conditions who underwent standard medical checkups during the same period were selected as the control group. Serum levels of IL-2, IL-4, IL-17A, TNF-a, IFN-g, and LIF were measured through ELISA. All participants in the AML group were followed up for three years. Based on the European Leukemia Network (ELN) genetic risk stratification criteria, patients were categorized into favorable, intermediate, and adverse prognosis subgroups. Inflammatory marker profiles were then compared among these subgroups.

**Results:**

Compared to healthy individuals, the AML group presented significantly increased serum concentrations of IL-4, IL-17A, and TNF-a, while levels of IL-2, IFN-g, and LIF were significantly decreased (P &lt; 0.05). Upon stratifying patients by prognostic classification, those in the favorable and intermediate prognosis categories exhibited notably lower IL-4, IL-17A, and TNF-a levels relative to the poor prognosis group (all P &lt; 0.05). In contrast, levels of IL-2, IFN-g, and LIF were notably elevated in the subgroup with favorable prognostic profiles (all P &lt; 0.05). Additional analysis of cytokine expression indicated that increased levels of IL-4, IL-17A, and TNF-a were linked to worse 3-year survival performance (P &lt; 0.05). Conversely, higher expression of IL-2, IFN-g, and LIF was significantly associated with better long-term survival outcomes relative to patients with reduced expression levels (P &lt; 0.05).

**Conclusions:**

The abnormal levels of IL-4, IL-17A, TNF-a, IFNg and LIF in patients with acute myeloid leukemia are increased, while the abnormal levels of IL-2, IFNg and LIF are decreased, which has certain guiding effect on prognosis assessment and can be used as auxiliary indicators for prognosis assessment of AML.

## Abstract (sr)

**Uvod:**

Cilj je bio da se proceni značaj inflamatornih faktora u predviđanju ishoda kod osoba sa dijagnozom akutne mijeloidne leukemije.

**Metode:**

U periodu od jula 2017. do decembra 2019. godine, ukupno 100 pacijenata sa nedavnom dijagnozom akutne mijeloidne leukemije (AML) regrutovano je sa Hematološkog odeljenja Centra za rak naše bolnice i raspoređeno u AML grupu. Pored toga, 60 osoba bez osnovnih zdravstvenih stanja koje su bile podvrgnute standardnim medicinskim pregledima tokom istog perioda odabrano je kao kontrolna grupa. Nivoi IL-2, IL-4, IL-17A, TNF-a, IFN-g i LIF u serumu mereni su ELISA testom. Svi učesnici u AML grupi praceni su tri godine. Na osnovu kriterijuma stratifikacije genetskog rizika Evropske mreže leukemije (ELN), pacijenti su kategorisani u podgrupe povoljne, srednje i nepovoljne prognoze. Zatim su upoređeni profili markera zapaljenja među ovim podgrupama.

**Rezultati:**

U poređenju sa zdravim osobama, grupa AML je imala značajno povecane serumske koncentracije IL-4, IL-17A i TNF-a, dok su nivoi IL-2, IFN-g i LIF značajno smanjeni (P &lt; 0,05). Nakon stratifikacije pacijenata prema prognostičkoj klasifikaciji, oni u kategorijama povoljne i srednje prognoze su pokazali značajno niže nivoe IL-4, IL-17A i TNF-a u odnosu na grupu sa lošom prognozom (svi P &lt; 0,05). Nasuprot tome, nivoi IL-2, IFN-g i LIF su bili značajno povišeni u podgrupi sa povoljnim prognostičkim profilima (svi P &lt; 0,05). Dodatna analiza ekspresije citokina ukazala je na to da su povecani nivoi IL-4, IL-17A i TNF-a povezani sa lošijim performansama u trogodišnjem preživljavanju (P &lt; 0,05). Suprotno tome, veca ekspresija IL-2, IFN-g i LIF bila je značajno povezana sa boljim dugoročnim ishodima preživljavanja u odnosu na pacijente sa smanjenim nivoima ekspresije (P &lt;0,05).

**Zaključak:**

Abnormalni nivoi IL-4, IL-17A, TNF-a, IFNg i LIF kod pacjenata sa akutnom mjeloidnom leukemijom su povecani, dok su abnormalni nivoi IL-2, IFNg i LIF smanjeni, što ima određeni uticaj na procenu prognoze i može se koristiti kao pomocni indikator A prognoze.

## Introduction

Acute myeloid leukemia (AML) is a common hematological malignancy characterized by uncontrolled proliferation and accumulation of undifferentiated myeloid cells within the bone marrow. These malignant cells can also infiltrate peripheral organs, including the spleen and thymus [1] [2]. Chemotherapy and allogeneic bone marrow transplantation remain the primary treatments for AML, yet the outcomes are still unsatisfactory [3]. Inflammatory factors play a role in promoting or inhibiting tumor in a paracrine form. In recent years, many scholars have studied microinflammatory homeostasis in leukemia patients. Research indicates that inflammatory cytokines are linked to clonal evolution and the advancement of AML [4], and further studies have shown that inflammatory cytokines have also been shown to be associated with fatigue, negative symptoms, and impaired quality of life (QoL) in AML patients [5] [6]. To date, comprehensive global investigations focusing on the early prognostic assessment of acute myeloid leukemia patients through multiple inflammatory biomarkers remain scarce. Therefore, this study aims to recruit a specific cohort of acute myeloid leukemia patients, assess their inflammatory markers, and investigate the relationship between inflammation and disease progression. The goal is to generate evidence for early-stage assessment of therapeutic response and prognostic trends, thereby supporting the development of personalized treatment strategies.

## Materials and methods

### Research object

A total of 100 patients with a recent diagnosis of acute myeloid leukemia were recruited between July 2017 and December 2019 from the Cancer Center's Hematology Department at our institution. The study population included 56 men and 44 women, ranging in age from 18 to 80 years, with an average age of 54.61 ± 15.43 years. Sixty healthy subjects who underwent physical examination in the physical examination center of our hospital during the same period were selected as the control group, including 33 females and 27 males, aged 20c78 years, with an average age of (53.52±15.33) years. Inclusion criteria: AML group: (1) First visit and meet the diagnostic criteria for acute myeloid leukemia [7]; (2) Age 18 years old; (3) The patient was diagnosed for the first time and did not receive any antitumor therapy before admission; (4) The patient gives informed consent and signs the consent form. Control group: (1) Healthy subjects without leukemia; (2) Age 18 years old; (3) Informed consent was obtained from the patient in writing. Exclusion criteria: (1) complicated with serious diseases of heart, brain, liver, lung, kidney and other important organs; (2) Combined with other malignant tumors; (3) Clinical data are incomplete.

### Methods

### Collection of clinical data of patients

General data of patients were collected through the medical record system of our hospital, including gender, age, height, weight, peripheral blood primitive cell proportion, bone marrow primitive cell proportion, immune typing, chromosome, fusion gene, gene mutation, treatment plan, and the recurrence and death time of patients were recorded through follow-up.

### Detection of serum Inflammatory factors

At the initial appointment, 3 mL of fasting venous blood was drawn and spun using a high-speed centrifuge (Eppendorf, Germany) at 3200 revolutions per minute. Serum samples were separated and obtained after centrifuging for 15 min. Corresponding ELISA kit was used for detection. The main detection indicators were as follows: (1) Interleukin (IL-2, IL-4, IL-17A) (2) Tumor necrosis factor a (TNF-α); (3) Interferon y (IFN-γ); (4) Leukemia inhibitory factor (LIF).

### Follow-up

Patients were followed up until December 30, 2022, with the end point of follow-up being death or disease progression, and 3-year survival was recorded. According to the 2017 European Leukemia Network (ELN) guidelines, patients in the AML group were divided into good prognosis group, medium prognosis group and poor prognosis group according to genetic risk stratification [8].

### Statistical analysis

All statistical procedures were conducted using SPSS software (version 22.0). The Kolmogorov-Smirnov test was applied to determine whether continuous data followed a normal distribution, and Levene's test was used to verify variance homogeneity. For data exhibiting normality and consistent variance, results were presented as mean ± standard deviation (x̄ ± s), and comparisons between groups were performed using one-way AN OVA. In cases where normality or homogeneity assumptions were not met, outcomes were summarized as medians along with interquartile ranges (25th-75th percentiles), and group differences were assessed via the Mann-Whitney U test. Categorical variables were reported in terms of frequency and proportion, and comparisons between independent groups were carried out using the chi-square test. A two-tailed P value below 0.05 was considered indicative of a statistically meaningful difference.

## Results

### Comparison of circulating inflammatory cytokine levels between AML patients and healthy controls

The detailed comparison is shown in Table 1. The AML group exhibited higher concentrations of IL-4, IL-17A, and TNF-α in circulation compared to healthy individuals (P < 0.05). Conversely, IL-2 levels were notably diminished in patients with AML. Although IFN-γ and LIF exhibited variable expression patterns, both cytokines differed meaningfully between the two study groups (P < 0.05). A comprehensive breakdown of the data is presented in Table 1.

**Table 1:** Comparison of serum inflammatory factors between the AML group and the control group.

Index	AML group (n = 100)	Control group (n = 60)	*t*	*P*
IL-2 (ng/mL)	19.72±8.04	39.62±3.52	18.138	0.000
IL-4 (pg/mL)	7.99±3.40	1.68±0.17	14.358	0.000
IL-17A (ng/mL)	16.19±3.38	9.56±1.03	14.782	0.000
TNF-α (ng/mL)	2.66±0.59	1.04±0.21	20.484	0.000
IFNγ (pg/mL)	24.91 ±10.33	56.98±5.36	22.295	0.000
LIF (ng/mL)	46.06±17.98	103.69±10.87	22.469	0.000

### Comparison of various indexes in patients with different prognosis

These differences among prognostic subgroups are summarized in Table 2. Patients in the intermediate and favorable prognosis groups exhibited significantly lower serum concentrations of IL-4, IL-17A, TNF-α, IFN-γ, and LIF compared to those in the poor prognosis group (all P < 0.05). Conversely, levels of IL-2, IFN-γ, and LIF were markedly elevated in both subgroups (all P < 0.05). Specifically, the good prognosis group demonstrated a pronounced decrease in IL-4, IL-17A, TNF-α, IFN-γ, and LIF, along with a significant increase in IL-2, IFN-γ, and LIF levels (all P < 0.05). Detailed comparisons are presented in Table 2.

**Table 2:** Comparison of serum inflammatory factors between the AML group and the control group.

Index	poor prognosis group<br>(n=42)	Medium prognosis<br>group (n=36)	good prognosis group<br>(n=22)	F	P
IL-2 (ng/mL)	12.42±1.36	20.34±2.09	32.62±3.54	583.031	0.000
IL-4 (pg/mL)	11.53±1.53	6.56±0.64	3.57 ±0.36	442.356	0.000
IL-17A (ng/mL)	19.61 ±1.62	14.36±1.53	12.67±1.64	174.325	0.000
TNF-α (ng/mL)	3.25±0.32	2.41 ±0.22	1.95±0.18	206.205	0.000
IFNγ (pg/mL)	14.96±1.52	26.87±2.67	40.68±4.65	593.340	0.000
LIF (ng/mL)	27.56±4.71	52.86±5.44	70.26±7.36	455.028	0.000

### Comparison of survival time of different Inflammatory Indicators

Based on inflammatory marker expression, participants were categorized into groups with high or low expression profiles. Analysis of survival trends revealed that individuals exhibiting elevated levels of IL-4, IL-17A, and TNF-α had significantly worse 3-year survival outcomes than those with lower expression levels (P < 0.05). In contrast, higher levels of IL-2, IFN-γ, and LIF were linked to improved survival within the same timeframe (all P < 0.05). A summary of these results can be found in Table 3.

**Table 3:** Comparison of 3-year survival rates for different inflammatory indicators.

index	3-year survival rate (%)	χ^2^	P
IL-2 high expression group (n=45)	28 (62.22)	12.334	0.000
IL-2 low expression group (n = 55)	15 (27.27)		
IL-4 high expression group (n=49)	9 (18.37)	23.785	0.000
IL-4 low expression group (n = 51)	34 (66.67)		
IL-17A high expression group (n = 54)	14 (25.93)	13.963	0.000
IL-17A low expression group (n =46)	29 (63.04)		
TNF-α high expression group (n=45)	6 (13.33)	29.379	0.000
TNF-α low expression group (n = 55)	37 (67.27)		
IFNγ high expression group (n = 50)	33 (66.00)	21.583	0.000
IFNγ low expression group (n = 50)	10 (20.00)		
LIF high expression group (n=47)	30 (63.83)	15.698	0.000
LIF low expression group (n = 53)	13 (24.53)		

## Discussion

Acute myeloid leukemia (AML) represents the most prevalent form of acute leukemia among adults, comprising approximately 80% of cases. However, the 5-year overall survival remains low, with only 40% of patients surviving beyond this period [8] [9]. This disease poses a serious threat to the health and survival of affected individuals. Although research on AML continues to deepen, its pathogenesis is still not fully understood. AML is highly heterogeneous, and the survival time of different patients varies greatly. Therefore, finding effective indicators to effectively predict the prognosis of patients is of great clinical significance for timely adjustment of treatment plans to improve the prognosis of patients [10].

In a paracrine form, inflammatory factors in the body participate in the tumorigenesis process with different functions. Inflammatory factors refer to a collection of polypeptide substances that regulate cells, such as interleukin, growth factor, cell stimulating factor, interferon, and tumor necrosis factor. In recent years, many scholars have studied microinflammatory homeostasis in leukemia patients. For example, Dong et al. [11] discovered that patients with acute leukemia had elevated serum levels of TNF-α and IL-8 compared to healthy individuals, and these two inflammatory factors could effectively show the disease progression of patients with acute leukemia [10] [11]. However, existing studies on the early assessment of treatment effectiveness and disease outcomes in AML patients— especially concerning the involvement of inflammatory markers—remain limited. This gap underscores the importance and relevance of the present study. In this study, six typical inflammatory factors, IL-2, IL-4, IL-17A, TNF-α, IFN-γ and LIF, were selected to further clarify the role of these six substances in the prognosis assessment of AML patients. Firstly, the serum levels of IL-2, IL-4, IL-17A, TNF-α, IFNγ and LIF were compared between AML patients and healthy controls. The study found that AML patients had significantly elevated levels of IL-4, IL-17A, and TNF-α, while levels of IL-2, IFN-γ, and LIF were significantly decreased compared to the control group (P < 0.05). The concentrations of IL-2, IFN-γ, and LIF were significantly reduced, and the differences reached statistical significance (P < 0.05). This is because IL-2, a member of the chemokine family, can promote the proliferation and functional differentiation of T cells, B cells, and natural killer (NK) cells, enhance both cellular and humoral immune responses, support the maturation of monocytes, and is often suppressed during the development of leukemia. TNF-α is beneficial to the body and has anti-tumor effects, but it has negative effects such as damaging the body when it excessively increases or its related cytokines are imbalanced during the onset of leukemia. IFN-γ can activate macrophages and have anti infective function. Scholars have reported that it plays an anti leukemia role by promoting Th1/Th2 balance in patients, so its level changes have high clinical value in evaluating the condition of leukemia patients [12]. IL-4, secreted byTh2 lymphocytes, is critically involved in driving the proliferation of activated B and T lymphocyte subsets. The differentiation of CD4+ T cells into the Th2 lineage is vital for regulating adaptive immune responses and supporting robust humoral immunity [13].

IL-17A is expressed in CD4+ T cells, and the protein induces IL-6 in fibroblasts. Subsequently, more researchers found that IL-17A is the main carrier of communication between T cells and the hematopoietic system, and overexpression in vivo can also lead to the formation of extramedullary granulocytes, which has been confirmed to be associated with various chronic inflammation and AML [14] [15]. LIF is a cytokine with various roles, primarily used to keep embryonic stem cells in an undifferentiated state, which is low in expression in children with acute leukemia [16]. This study also further confirmed its low expression and significant inhibition in adult AML. In order to further evaluate its role in prognostic assessment, this study stratified the AML group of patients according to genetic risk according to the 2017 European Leukemia Network (ELN) guidelines: According to ELN risk classifications, inflammatory marker levels were compared across favorable, intermediate, and adverse prognosis subgroups. Findings revealed that IL-4, IL-17A, and TNF-α concentrations were lowest in the favorable outcome group, whereas IL-2, IFN-γ, and LIF levels were highest, suggesting their potential association with favorable prognosis. At the final stage of the analysis, participants were grouped by cytokine expression into high- and low-expression categories. Individuals with elevated IL-4, IL-17A, and TNF-α expression exhibited significantly decreased 3-year survival outcomes in comparison to those with lower cytokine expression (P < 0.05). On the other hand, elevated levels of IL-2, IFN-γ, and LIF were linked to a notably better 3-year survival rate (all P < 0.05). This clearly demonstrated that alterations in IL-2, IFN-γ, and LIF levels can effectively forecast patient outcomes and serve as reliable indicators for prognosis prediction.

In conclusion, patients with acute myeloid leukemia exhibit elevated levels of IL-4, IL-17A, and TNF-α, while IL-2, IFN-γ, and LIF are significantly reduced compared to healthy individuals. However, higher intra-cohort expression levels of IL-2, IFN-γ, and LIF are associated with favorable prognosis and improved 3-year survival. These cytokine profiles may serve as valuable supplementary biomarkers for prognostic evaluation in AML.

## Dodatak

### Conflict of interest statement

All the authors declare that they have no conflict of interest in this work.
